# Veganism and paediatric food allergy: two increasingly prevalent dietary issues that are challenging when co-occurring

**DOI:** 10.1186/s12887-020-02236-0

**Published:** 2020-07-10

**Authors:** Jennifer L. P. Protudjer, Andrea Mikkelsen

**Affiliations:** 1 Department of Pediatrics and Child Health, University of Manitoba, Winnipeg, Canada; 2 George and Fay Yee Centre for Healthcare Innovation, Winnipeg, Canada; 3 Children’s Hospital Research Institute of Manitoba, Winnipeg, Canada; 4 Department of Food and Human Nutritional Sciences, University of Manitoba, Winnipeg, Canada; 5 Centre for Allergy Research, Karolinska Institutet, Stockholm, Sweden; 6 Paediatric Primary Health Care Clinics of Västra Götaland, Västra Götaland, Sweden; 7 Research and Development Primary Health Care, Gothenburg and Södra Bohuslän, Region Västra Götaland, Sweden; 8 Institute of Medicine, Department of Public Health and Community Medicine, Sahlgrenska Academy, University of Gothenburg, Gothenburg, Sweden

**Keywords:** Food allergy, Nutrition, Pediatrics, Vegan

## Abstract

Vegan diets – defined as the exclusion of all foods of animal origin from the diet- are becoming popular. In recent years, the prevalence of food allergy has also increased, and disproportionately affects children. When vegan diets and food allergy co-occur, this combination can be challenging and pose risks of nutritional deficiencies, particularly during childhood. In this paper, we aim to summarise the major concerns regarding vegan diets and food allergy, review the literature on this topic, and provide some suggestions for healthcare providers, particularly dietitians and nutritionists, who work with food allergic, vegan patients and their family. When working with this patient population, a regular and complete medical nutrition history, including screening for any possible nutritional deficiencies, is warranted. Likewise, the routine tracking of serum markers (especially iodine, iron, zinc, calcium, Vitamins B12, D, B2, and A, selected n-3 fatty acids and protein, which are more abundant in animal vs. plant foods) and symptoms of co-morbid diseases, including asthma, is important, as comorbid diseases may increase energy and nutrient requirements. For infants and children, anthropometry ought to be tracked longitudinally at regular intervals to identify any deviations from the child’s previous growth pattern, and to accommodate any increased requirements for growth and development. Correct diagnoses, education and allergy management must be disseminated to the family in a clear and appropriate manner. Children with allergy may have increased nutritional needs due to comorbidity. This is complicated by coincident food allergy and vegan diet as both impose diet restrictions (limiting sources of important nutrients, need for dietary variety and/or increased consumption due to reduced bioavaliability).

## Introduction

Vegan diets – defined as a diet that excludes all animal products, including meat, poultry, fish, molluscs and crustaceans, cow’s milk, eggs, gelatin and honey [1, 2], aside from breastmilk are increasingly popular. In the United States, 3.4% of the total population state that they follow a vegan diet, with an estimate of 1% of children 8–18 years of age. Data from Europe support slightly lower rates, at approximately 1% of the population, with estimates unknown for mothers and children [3, 4].

In recent years, the prevalence of food allergy has increased in many parts of the world [5], as have hospitalisations due to food allergic reactions [6]. Prevalence estimates range widely, with an accepted estimate of probable Immunoglobulin-E mediated food allergy in high income nations between approximately 2 and 7% [7, 8]. Over the past three to four decades, food allergy prevalence has increased sharply, with some recent data pointing toward a plateau. In an attempt to minimize the possibility of an adverse, and potentially severe or even fatal reaction, food allergy necessitates exclusion of the allergenic food [5].

Guidelines for vegan diets amongst paediatric patients issued by national food administrations and/or nutrition societies in several countries, as well as international expert groups (including the European Society of Paediatric Gastroenterology, Hepatology and Nutrition; [ESPGHAN] [9]; Dietitians of Canada [10], and the American Academy of Nutrition and Dietetics [10]) now endorse well-planned and carefully monitored vegan diets. Likewise, careful monitoring of food allergy is necessary to ensure the nutritional needs of affected children are met [11]. At the intersection of veganism and food allergy, there are even greater concerns related to adequate micro- and macronutrient intakes that warrant careful consideration and monitoring. In this paper, we aimed to summarise the major concerns regarding vegan diets and food allergy, review the literature on this topic, and provide some suggestions for healthcare providers, particularly dietitians and nutritionists, who work with a food allergic, vegan patient.

### Vegan diets: health benefits and risks

Previously, we have referred to a definition of vegan diet by what is eliminated. However, a vegan diet can also be described as rich in a wide variety of foods: whole grains, legumes, vegetables, fruits, nuts, seeds, vegetable fats, herbs and spices [3]. The high amount of macro- and micronutrients, as well as a wide variety of carbohydrate types seems to enhance the development of a diverse intestinal flora [12, 13], of increasing interest in disease prevention [14]. Benefits of, and reasons for adopting a vegan diet range widely, and include animal welfare [15], health benefits [15–17], personal well-being [15], improvement of disease symptoms [18], increased meat prices [19] and environmental concerns [20, 21].

Recently, vegan diets were found to be an effective means of prevention and treatment of cardiometabolic disease [22, 23], with a decreased risk up to 40% of associated diseases. Moreover, the risk of metabolic syndrome and type 2 diabetes may be decreased by 50%, and, if well planned, can assist in reversing atherosclerosis, and reducing blood lipids and blood pressure. These health benefits may result, in part, from the observation that, compared to non-vegans, those following a vegan diet have a lower body mass index (BMI) and waist circumference. But there are some exceptions. In a recent meta-analysis, compared to a typical Taiwanese diet, vegan diets are not associated with improved cardiometabolic outcomes or lower BMI [23, 24].

However, potential vegan diet pitfalls exist, particularly amongst children. Young children may benefit from a variety of plant-based proteins during small and frequent meals to allow for consumption of a large volume of foods [3]. Whereas many of guidelines support vegan diets for infants and children [2, 12, 13, 25, 26], there are some exceptions to these guidelines [27].

### Vegan diets and macronutrient deficiencies

Much of the literature on vegan diets and nutritional outcomes is based on adult populations [14, 22, 28–33]. Less is known about vegan diets amongst infants and children. Thus, these age groups need to be carefully and routinely monitored, if following a vegan diet [34]. Infants and children have higher energy and nutrient needs, which may be difficult to achieve on a restricted diet. Recent data support that children to age 3 years following a vegan diet consume adequate energy intake, but compared to omnivore children, significantly more carbohydrates (and fibre) and significantly less protein [35].

Protein requirements in vegan diets are met mainly through vegetables, tofu, beans, whole grains, nuts and seeds. In order to achieve sufficient amounts of essential amino acids, daily varied consumption is recommended [36]. As the above-mentioned foods often contain considerable amounts of fibre and anti-nutritional factors, i.e. substances that prevent optimal absorption of specific nutrients, protein intakes are recommended to be increased by 10% during periods in life when protein needs are higher. Two of these periods are infancy and childhood [3]. Protein needs can be met in a well-planned vegan diet, provided that the caloric need is met. For this reason, vegan protein-rich alternatives with less fibre, such as tofu and seitan, may be preferable since these foods usually result in high satiety and might support appropriate protein intake [37, 38]. First results from a Swedish trial of infants randomized to a diet with decreased protein intake, compared to a traditional Nordic diet has, thus far, shown no group differences in growth or iron status at 9 months of age [39]. Forthcoming results will glean further insight into the role of decreased protein intake, compared to a traditional Nordic diet, on anthropometric outcomes, metabolic and inflammatory biomarkers, as well as the gut microbiome [40].

These data notwithstanding, there are case reports of alarming nutritional deficiencies as a consequence in what has, sometimes been described as vegan diets. However, critical review of these reports can lead to the conclusion that these children often have very restricted vegan diets with limited caloric intake [41], or very limited food choices, for example extensive use of unfortified plant-based beverages [42]. In extreme situations, these restrictions may contribute to protein-calorie malnutrition [42], or kwashiorkor, in which the child consumes adequate calories but inadequate protein [43] as well as inadequate micronutrients [42]. Notably, these diets cannot be considered well-planned vegan diets, and any conclusions regarding vegan diets for children based solely on these reports are misleading [3].

### Vegan diets and micronutrient deficiencies

A poorly planned vegan diet can increase the risk of micronutrient deficiencies, particularly iodine, iron, zinc, calcium, Vitamin B12, Vitamin D, Vitamin B2, Vitamin A, n-3 fatty acids (docosahexanoic acid; DHA).

#### Iodine

Iodine deficiency, which is poorly studied in high income nations, has been described in vegan infants who are breastfed [44] or recently weaned [45]. In this instance, thyroid stimulating hormone levels should be monitored and, if necessary, treated appropriately. In a vegan diet, iodine requirements can be met with iodized salt or algal source supplements [3]. That said, it warrants mention that the amount of iodine in salt varies by country or region [46]. It also warrants mention that cases of iodine deficiency amongst milk allergic infants who receive inappropriate supplementation have been described. In a Norwegian study of 57 infants aged < 2 years, 30% had urine iodine concentrations lower than the World Health Organization cut-off values [47]. Amongst infants predominantly breastfed, this number doubled, to 58%. Notably, iodine deficiency was independent of poor growth status [47].

Infant foods should not have added salt [12]. As such, iodine requirements are better met through breastmilk. A recent study found that breastmilk iodine concentrations were not associated with pre-pregnancy weight, although maternal diet was not considered in the analyses [48]. In contrast, the Centers for Disease Control and Prevention advises that the recommended daily allowance is 290 μg iodine for breastfeeding women, and that a supplement is indicated for women who do not consume dairy products [49]. Alternatively, legally sold commercial infant formulas fortified with iodine are widely available, and are carefully regulated [50].

#### Iron

Typical vegan diets often include many iron-rich foods, including lentils, tofu (soy), chickpeas, nuts, seeds and grains. However, unlike iron from animal sources, termed heme iron, which is readily absorbed [51], non-heme iron from plants has poor bioavailability and lower absorption, due to high phytate and polyphenol levels [52]. Non-heme, plant-based sources of iron must also be consumed in larger portions than heme, animal-based sources of iron, to meet needs [53]. Vegans, as well as vegetarians, require 1.8 times the amount of dietary iron, compared to meat eaters, as a result of poorer bioavailability [54]. Vitamin C may enhance the absorption of non-heme iron [55]. However, many vegan sources of iron, particularly soy, nuts and sesame seeds, are also common food allergens. For children who are not allergic, iron-fortified foods, including packaged cereals, may be an additional source of iron. Through later infancy, iron deficiency is the most prevalent micronutrient deficiency, thus highlighting the importance of adequate iron intake [56]. Amongst the very young, infant formulas and cereals are main sources of dietary iron [57]. After age 12 months, iron-fortified formulas are positively associated with serum ferritin levels, independent of socio-economic status and other dietary sources of iron [56].

But, even in the absence of allergies to common iron-rich vegan foods, careful monitoring of these children may be advised. In a study of infants with cow’s milk allergy, but otherwise well, iron deficiency anaemia was identified in 5% of children [58]. More strikingly, a retrospective study from Taiwan provides evidence that the prevalence of cow’s milk protein allergy amongst children with iron-deficiency anaemia (serum ferritin < 12 ng/mL) [59] was at least double the prevalence that has been reported in the general Taiwanese population (13.7% vs. 3.4 to 7.7%) [59, 60]. Although the study of children with iron-deficiency anaemia was small (*N* = 51), it points toward an increased risk of iron-deficiency anaemia amongst young children with cow’s milk allergy. Notably, although these children were quite ill, as evidenced by failure to thrive, hypoalbuminemia and erosive and hemorrhagic colitis on colonoscopy, none had eosinophila in the lamina propria and all recovered within 7 months of cow’s milk elimination and iron supplementation [59].

#### Zinc

Zinc, like iron, has poor bioavailability, especially in a vegan diet [52]. Vegan sources of zinc include soy and other legumes, nuts, seeds and whole grains as well as fortified cereals. Due to the lower bioavailability of zinc in plant foods, vegans (as well as vegetarians) may require 1.5 times more zinc than meat eaters [61]. Notably, soaking, heating and fermenting zinc-rich plant foods appears to reduce the inhibitory effect of phytate, which contributes to greater absorption of zinc [62, 63].

Few studies have specifically examined zinc status in vegan vs. omnivore children. Authors of a 2014 review article reported no differences in serum zinc concentrations between young vegetarian, including those following a vegan diet, vs. omnivore children, although some differences may present in adolescence [52]. However, the same authors also note that zinc status amongst vegan children should be monitored, and if necessary, provided with zinc supplements [52]. Finally, as vegan sources of zinc typically include prepackaged, fortified breakfast cereals, legumes and soy, particular attention is warranted when working with vegan children who must avoid these foods due to allergy.

#### Calcium

Calcium is a vital mineral for bone mineral density development, but is predominantly obtained through dairy products which are notably absent from the vegan diet. In childhood, recommended intake of calcium is high in order to achieve a positive balance to promote growth. Notably, high calcium intake in western countries is correlated with higher risks of fractures [64]. Elsewhere, many food cultures rely on vegetables, legumes and cereals as the main source of calcium. For example, a study in China concluded that dairy products provided only 6.7% of dietary calcium [65]. This was also the case in Japan, with the addition of fish and shell-fish, and somewhat higher levels in urban areas where dairy consumption is increasing [66]. The increasing interest in other food cultures, alongside with increased global availability of novel foods may provide useful sources of calcium. However, the allergenicity of uncommon foods may be poorly known and inclusion of many novel foods need careful assessment and consideration [67]. In a Dutch study, adolescents who had followed a vegan-type diet in early life had significantly lower bone mineral density in adolescence [68]. These differences were not explained by current calcium intake, suggesting that early life is a critical window for bone mineral density development. This study was performed approximately 20 years ago. Since that time, numerous calcium-fortified food products and plant-based milks are now available. However, caution is warranted to ensure that young vegans consume adequate levels of calcium to maximise bone mineral density. This need for caution is underscored in a recent meta-analysis, in which, compared to omnivores, vegetarians and vegans had lower bone mineral density, and vegans also had higher rates of fractures [69]. In addition to the numerous calcium fortified alternatives for dairy products available today, calcium is also found in most green leafy vegetables, preferably those with low levels of oxalate as it inhibits absorption, sesame seeds, almonds and dried figs [3, 70]. Finally, if calcium requirements are not met, supplementation may need to be considered, eventually in combination with Vitamin D, which promotes calcium absorption [71].

#### Vitamin B12

Vitamin B12 is perhaps the most common concern for those following a vegan diet, as it is found almost exclusively in food of animal origin [72]. Infants born to mothers with inadequate Vitamin B12 status during pregnancy are at risk of developing various hematological, neurological and developmental problems [73–75]. As well, vegan mothers who breastfeed their infants should receive Vitamin B12 supplementation [76]. It has been suggested that Vitamin B12 supplements or fortified foods should be an ongoing part of the diet [77]. Whereas supplemental Vitamin B12 may be an option for those following a plant-based diet, it warrants mention that approximately only 1% of oral Vitamin B12 supplements cross the intestinal barrier [78], thereby necessitating substantial and sustained need for Vitamin B12 supplements. Many plant-based beverages are fortified with Vitamin B12, and which are palatable and readily incorporated into the diet. Notably, most plant-based “milks” are not intended as a primary beverage until at least 2 years of age, as they are often low in calories and/or fat, and may not be (adequately) fortified to meet infants’ needs [79]. For children < 2 years of age with greater needs for growth and neural development, and disproportionately impacted by food allergy, [5] breastfeeding should be promoted or appropriate formula used [9]. Children and adolescents following a vegan diet show significantly lower B12 intake and serum vitamin B-12 concentration levels compared to omnivore peers [80, 81]. However, levels were not always deemed as low according to guidelines [82].

#### Vitamin D

Despite lack of conclusive evidence, most trials and interventions indicate that vitamin D_3_ of animal origin is more effective than vitamin D_2_ of plant source [83]. Humans obtain much of their Vitamin D through sun exposure. Dietary intake of Vitamin D is limited to animal products, such as fatty fish. In western countries, as many as 40% of children are Vitamin D deficient in the wintertime [84, 85]. Although it could be suggested that this is due to limited sun exposure, these estimates are just as high in southern European countries, such as Greece, where sun exposure throughout the year is high [86].

Both vitamin D_2_ and D_3_ have been identified in a lichen species (*Cladina* arbuscula) suggesting a potential vegan source candidate [87]. However, studies exploring its efficacy are currently lacking.

#### Vitamin B2

Vitamin B2, or riboflavin, is necessary for the metabolism of amino acids and carbohydrates, and development of the nervous system. Major dietary sources include milk, eggs and some meats, which do not form part of the vegan diet, as well as leafy greens, fortified grains, nuts and soy [88]. As riboflavin is abundant in many plant products, vegan children often have adequate intakes of this vitamin [34].

#### Vitamin a

Vitamin A, commonly found in fortified foods and beverages, including milk, as foods of animal origin (e.g. cod liver oil, eggs) and leafy greens rich in beta-carotene (e.g. kale, spinach). Whereas vegan diets may be rich in the latter, both male and female vegans nonetheless had significantly lower Vitamin A intakes than individuals following a more traditional (i.e. unrestricted) diet. Vegan intakes were also below nutritional recommendations [89].

#### N-3 fatty acids (DHA)

Omega-3 fatty acids demand special attention in the vegan diet, more so during pregnancy, lactation, infancy and childhood. Inclusion of vegan, omega-3-containings foods, such as walnuts, ground chia seeds and ground flaxseed, is advisable [3]. At the same time, it warrants mention that these foods are also high in alpha linolenic acids. Although the body can convert alpha linolenic acid into DHA and eicosapaentinoic acid, this process is not efficient [3]. Moreover, there have been concerns raised about the processing of flaxseed [90, 91]. At present, the safe amount of ground flaxseed is not well known and caution is advised [91, 92]. Algal-oil supplements have been found to be as effective as fish-oil based alternatives [93, 94], even when cognitive outcomes have been studied [95]. Alternatively, supplementary sources of preformed DHA should be considered [3].

### Special nutritional considerations in food allergic individuals

An important reminder on the role of the allergy team is to help identify the specific foods to be eliminated from the diet and preventing further avoidance expanding to whole food groups which limits the diet unnecessarily. For example, cooked vegetables and fruits, as well as roasted nuts may sometimes be consumed by those with oral pollen related food syndrome (or oral allergy syndrome) due to birch-, mugwort- or other pollen allergies. Likewise, patients with soy allergy may tolerate other beans and/or lentils within the wide pulse family. Additionally, vegan patients with allergy to peanuts and tree nuts may consider other nuts and seeds as good alternatives. Professional advice is warranted to assist patients and prevent the unnecessary exclusion of important sources of protein and nutrients in a vegan diet [96, 97].

Compared to non-food allergic children, allergic children consume significantly less calcium and protein, and are more likely to have diets that are deficient in essential fatty acids [98]. These nutritional inadequacies are associated with other concomitant micronutrient deficiencies [99], have direct impacts on bone mineral density and physical growth [99], and may impair learning [100]. Such differences also appear to exist between children with different types of allergy, suggesting that the food to which a child is allergic, and thus must eliminate from his or her diet, also needs to be considered. For example, children with cow’s milk allergy had significantly lower calcium intakes than children with non-cow’s milk food allergy [101]. Importantly, any food allergy (not limited to only cow’s milk allergy) in childhood may predict non-significant differences in calcium intake in adolescence [102]. Likewise, in observational studies, both zinc [71] and iodine deficiencies [103, 104] have been noted for cow’s milk allergic children, even if the child was taking a vitamin/mineral supplement [105].

The few previous studies on the associations between anthropometry and food allergy have been cross-sectional [106, 107] and thus report on anthropometrical differences between children with or without food allergies, rather than growth*.* Nonetheless, these studies provide evidence that both height and weight are negatively impacted by the presence of food allergy. For example, American children with food allergies were found to be significantly shorter and lighter, but not different in terms of body mass index (BMI; (weight (kg)/height (metres^2^))) [108], than non-food allergic peers. Notably, many of these differences were attenuated upon consideration to type of health insurance, and also varied by age group [106]. Likewise, British researchers reported that both underweight and short-for-age affected approximately 10% of food allergic children, compared to World Health Organization standards [107]. These differences became even more pronounced amongst children with multiple food allergies.

Whereas the above studies provide evidence that children with food allergy are more likely to be underweight, in at least one study, British researchers found that children with food allergy were significantly more likely to be overweight or obese than their non-allergic peers [107]. For children with food allergy and who follow a vegan diet, the risks of overweight and obesity are likely to be low, provided that they follow a well-planned, nutrient rich diet. However, this British study provides evidence that overweight and obesity can nonetheless occur.

Even in the absence of nutrient deficiencies, height, weight, weight-for-age, and weight-for-height may be lower by as much as two standard deviations [107]. Lumbar spine bone mineral density is also significantly lower in children with cow’s milk allergy compared to children with non-cow’s milk food allergy [101]. Such differences are indicative of moderate malnutrition, and are thus clinically relevant issues that warrant immediate attention [107]. These previous studies were performed in high-income countries, but which lack national school meal programs or, in some cases, widely subsidised prescriptions for allergic children.

Finally, vegan diets have been associated with a higher odds of atopic dermatitis [109]. Thus, food allergic individuals following a vegan diet ought to be cautioned about this possibility. In contrast, patients with asthma who followed a vegan diet for 1 year had dramatically improved clinical biomarkers [110].

Given the restrictions associated with a plant-based diet, even greater attention is warranted when those following a vegan diet also have additional restrictions resulting from food allergy. Recently, American authors reported that people with asthma, another allergic disease but which does not mandate dietary restrictions, commonly turned to YouTube for information on vegan diets [111]. However, such information was frequently deemed to be of poor quality [111].

In light of the above-described concerns, it is unsurprising that numerous nutrition and dietetic societies advise that vegan diets should only be used under appropriate medical or dietetic supervision with expertise in food allergy. Moreover, parents should understand the serious consequences of failing to follow advice regarding supplementation of the diet [9]. These guidelines reflect a shift from a decade ago, when total avoidance of a vegan diet in infants and young children was recommended [112]. Failure to follow dietary advice may compromise both nutrition and growth. These conditions become even more important when the diet is further limited by food allergy.

Soy, peanuts, tree nuts and wheat are three of the six most usual foods IgE-mediated food allergies [113] and soy the second most common eliciting food in non-IgE mediated food allergies [114, 115] further restricting the choices for vegans. Special attention has to be paid to the amount, as well the quality of the protein in the vegan diet [3, 5, 38, 114, 116].

Of additional concern is the volume of food (i.e. portion size) needed to achieve recommended/appropriate levels of energy and nutrients [3, 5, 38, 114, 116]. As such, one cannot dismiss the possibility that children with food allergy and who have allergic conditions face particular challenges meeting their nutrient and energy demands through a vegan diet.

Protein is the macronutrient of concern in this unique population. But, many plant-based, protein rich foods are also common allergens. Soy-based formulas are a common alternative to cow’s milk formulas, for both vegan infants and those with cow’s milk allergy. However, soy-based formulas should not be prescribed to prevent the development of food allergy [117, 118]. Likewise, soy formula is not recommended for infants aged < 6 months, due to concerns of lower rates of absorption of minerals and trace elements due to the phytate content in soy [119]. Nuts are also a key source of protein and fat, but must be avoided by those with a nut allergy. Likewise, almond milk, a common plant-based alternative to cow’s milk, must be avoided by those with almond allergy. Yet, almond milk is richer in calcium and fat, yet low in calories compared to other plant-based milks [120]. For vegans with a nut allergy, nut avoidance can be particularly difficult [121]. It is recommended that specific nuts to which an individual is not allergic should be introduced. However, caution is warranted with pre-packed snacks with precautionary allergen labelling [122].

Some groups have greater energy and nutrient demand. For infants, children and athletes who follow a vegan diet and also have food allergy, caution is warranted to ensure optimal intakes. Achieving greater intake may be challenging due to the portion size needed to achieve recommended/appropriate levels of energy and nutrients [3]. The challenge is higher for infants and children who generally have higher needs due to growth [107, 123], as well as in certain atopy, e.g. atopic dermatitis [124]. Recently, biologically negligible, but statistically significant differences in resting energy expenditure, anthropometry, and dietary intake in an Italian study of 30 children with food allergy matched to 31 healthy children were described [124]. In contrast, others have reported that sustained intestinal or skin inflammation due to allergies [125, 126] and avoidance diets [127–129] may contribute to impaired growth.

The above examples highlight the complexity of managing cross-reactions for those with food allergy, while limiting avoidance to only those foods that elicit symptoms. People with cross-reactions due to birch pollen allergy (i.e. those who test negative for the storage proteins Ara h 1, 2, 3 and 9 and positive for Ara h 8 for peanut and / or negative tests for Cora 8, 9 and 14 storage proteins and positive for Cor a 1 hazelnuts) are more likely to have non-life-threatening reactions [130]. When possible, it would be beneficial to test for reactivity to food components, rather than only food-specific IgE [130]. In the absence of the availability of such testing, SPT may be of some benefit only if patients complain of allergic type symptoms to both food types. Otherwise, patients may be encouraged to consume soy, lentils and chickpeas carefully (at first exposure) or *ad libidum* (if testing is negative, and/or if they are asymptomatic when consuming these foods). As these food types represent a major calorie- and protein source for those following a vegan diet, the elimination of these food types is strongly cautioned unless the peanut allergic patient has a confirmed allergy to soy, lentils and/or chickpeas.

In cases where a vegan patient is allergic to soy, the dietitian is encouraged to review common sources of soy in the diet, both in obvious forms, such as tofu and soy-based beverages, as well as in processed foods, which commonly contain soy lecithin. These patients should be considered particularly vulnerable for protein deficiencies, given than soy is a common, widely available and generally palatable source of protein in the vegan diet. True soy allergies, as confirmed by OFC, are uncommon [131]. Clinical experience suggests that many patients with soy allergy may tolerate soy lecithin, likely due to low antigenicity of the proteins in soy lecithin and soy oil [132]. The need of avoidance of soy lecithin seems to be reduced to a couple of case reports on children with severe soy allergy reacting to soy lecithin in medication [133, 134]. Further complicating the understanding of soy allergy is evidence that children may develop tolerance to soy, not so to peanut [135]. Taken together, these findings underline the importance of a careful diagnosis, and, when prudent, re-evaluation of soy allergy. Thus, the decision to include or exclude soy lecithin should be taken on case-by-case basis.

Patients with peanut and tree nut allergies, are less likely to achieve clinical tolerance than patients allergic to milk or egg [5, 136]. Pea protein is an increasingly widely available vegan protein, and may be a suitable alternative to soy. However, pea allergies have been documented [137], most commonly amongst those with allergies to other legumes.

Patients who are also allergic to wheat pose a challenge in that they are unable to consume wheat protein alternatives, including seitan, as well as more widely available vegan alternatives, such as wheat-based pastas or breads. These foods represent protein-, calorie- and convenience losses to the patient. Alternatives to consider are bean- and lentil-based pastas, as well as legumes in their original forms.

Pain noir, or buckwheat, is a common staple in parts of Asia and northern France, and is commonly found in noodles, and flour mixes originating from these regions, as well as many gluten free flour mixes in these regions and beyond. Buckwheat is botanically related to grasses, rather than wheat, and thus can be consumed freely by wheat allergy sufferers. However, cross-reactivity between buckwheat and peanut has been demonstrated [138].

### Specific recommendations when working with food allergic individuals

Suggestions for working with a food allergic, vegan patient and their family are provided in Table 1. In addition to regular support from health professionals, families may also wish to engage with their local patient organisations, as many of these organisations offer support groups. These, as well as patient and/or parental groups organised by healthcare have proven to be a good source of support and effective in encouraging families to take necessary actions [141], not less during the challenging process of re-introducing food when the allergy is out-grown [96, 151, 152] or any other underlining the importance of personalised/individualized management.

**Table 1:** Suggestions for working with a food allergic, vegan patient and their family

Suggestion	Reference
Caution parents of the need for professional dietary monitoring and potential need for supplementation to meet the needs of a growing child	[9]
Careful attention must be given to the amount and quality of dietary protein, particularly as many plant-based protein rich foods are common allergens	[3, 5, 38, 114, 116]
Counsel families that portion sizes needed to achieve recommended/appropriate levels of energy and nutrients through a plant-based diet are greater than a traditional diet	[3, 5, 38, 114, 116].
Caution is warranted with pre-packed snacks with precautionary allergen labelling	[122]
Food labels must be read every time a food is purchased, to reduce the risk of accidental ingestions and reactions	[139]
‘May contain’ labels to alert consumers to possible traces of allergens in a food product, although the amount of allergens in a given food varies widely, resulting in a need for consumer vigilance	[140]
Encourage cautious re-introduction of a food if a child outgrows an allergy	[141–143]
Encourage a diverse diet, as restrictive diets and selective eating may contribute to nutritional deficiencies	[127, 144–146]
Discuss any new food and environmental allergies, as cross-reactivity can occur	[147–149]
Educate families that some allergies can be influenced by season and climate	[150]

Given the restrictions associated with a plant-based diet, even greater attention is warranted when those following a vegan diet also have additional restrictions resulting from food allergy. Recently, American authors reported that people with asthma, another allergic disease but which does not mandate dietary restrictions, commonly turned to YouTube for information on vegan diets [111]. However, such information was frequently deemed to be of poor quality [111].

Finally, it warrants mention that those who follow a vegan diet are typically quite health conscious and engage in more physical activity compared to omnivores [153, 154], although this evidence is collectively inconclusive [155]. These characteristics need to be considered when interpreting the results of studies on health outcomes in vegan populations.

## Conclusions

Children with allergy may have increased nutritional needs due to comorbidity. This is complicated by coincident food allergy and vegan diet as both impose diet restrictions, thereby limiting sources of important nutrients, need for dietary variety and/or increased consumption due to reduced bioavailability.

## Abbreviations

BMI: Body mass index
DHA: Docosahexanoic acid
ESPGHAN: European Society of Paediatric Gastroenterology, Hepatology and Nutrition
IgE: Immunoglobulin E
OFC: Oral food challenge
SPT: Skin prick test
